# 

**Published:** 1883-10

**Authors:** 


### Ohio State Dental Society.

The eighteenth annual meeting of the Ohio State Dental Society will be held in the city of Columbus on Wednesday, October 31, at 10 o'clock, A.M.

This is a little more than a month earlier than the usual time of the meeting. This change was made with the expectation that the weather would be more pleasant than it has generally proved to be at the first of December, and there is but little doubt that this expectation will be realized.

We shall hope that on this account there will be a larger attendance than usual. Our State Society has not for the past two or three years been as largely attended as formerly, or as its friends desired.

It is to be hoped that all the members will be present, and plenty of material for new members. Let all come prepared to add to the interest and profit of the occasion.

If this Society is to sustain its reputation and keep pace with the societies of the neighboring States its members must put forth equal, if not greater, efforts than heretofore. Let this suggestion be borne in mind and acted upon.

A good programme is presented for discussion, upon each of which there should be several good papers.

The following are the subjects:

1. Etiology of Dental Caries.
2. Is Dental Decay Unitary or Multiple?
3. Dental Education.
4. Tin Foil as a Filling.
5. First Dentition.

---

Died at his home in Philadelphia, Sept. 11th, 1883, of general prostration and paralysis, Professor Thos. L. Buckingham.

Dr. Buckingham was one of the oldest dental practitioners in Philadelphia, and one of the oldest dental teachers in the country. He was one of the founders of the Pennsylvania Dental College, and was one of its Professors, from the beginning till the time of his death.

---

### State Board of Dental Examiners.

The annual meeting of this board for the examination of applicants will be held at the Neil House, in Columbus, at 12 o'clock on the last Wednesday (31st) of October.

All interested will make a note of this, and govern themselves accordingly and be promptly on hand at the designated time.

### CONTENTS.

### COMMUNICATIONS.

### THE DENTAL REGISTER,

A MONTHLY MAGAZINE DEVOTED TO THE INTERESTS OF THE

### DENTAL PROFESSION.

*Terms Reduced to* $2.00 *Per Year. Single Copies,* 25 *Cents.*

### EDITED BY PROF. J. TAFT, D.D.S.

—AND—

### Published by SPENCER & CROCKER, Ohio Dental and Surgical Depot.

The volume commences in January and closes in December.

The REGISTER being issued monthly is always fresh, therefore Indispensable to the practitioner who desires to keep posted up with the new inventions and improvements which are daily developed. Neither expense nor effort will be spared to give value and variety to its contents. Articles will be illustrated with well executed engravings whenever they will add to the interest or assist to explanation.

Its extensive circulation and frequency of issue make it one of the BEST DENTAL ADVERTISING MEDIUMS in the United States.

All subscriptions must begin with January or July.

Local or special notices, five lines or less, will be inserted for $3 each insertion; over five lines, 50 cts. per line.

All advertising bills payable in advance, or at furthest, after the issue of the first number containing the advertisement. All transient advertisements to be paid for in advance.

☞CONTRIBUTIONS SOLICITED.

Exclusively Editorial Communications should be addressed to the Editor.

The latest number of the REGISTER may be ordered with or without other goods at any time, and all letters should be addressed to

**SPENCER & CROCKER, Ohio Dental & Surgical Depot, Cincinnati, O.**

